# A Novel Paradigm Based on ST2 and Its Contribution towards a Multimarker Approach in the Diagnosis and Prognosis of Heart Failure: A Prospective Study during the Pandemic Storm

**DOI:** 10.3390/life11101080

**Published:** 2021-10-13

**Authors:** Radu-Stefan Miftode, Daniela Constantinescu, Corina Maria Cianga, Antoniu Octavian Petris, Amalia-Stefana Timpau, Adrian Crisan, Irina-Iuliana Costache, Ovidiu Mitu, Dana-Teodora Anton-Paduraru, Ionela-Larisa Miftode, Mariana Pavel-Tanasa, Petru Cianga, Ionela-Lacramioara Serban

**Affiliations:** 1 Department of Internal Medicine I (Cardiology), Faculty of Medicine, University of Medicine and Pharmacy “Gr. T. Popa”, 700115 Iasi, Romania; radu-stefan.miftode@umfiasi.ro (R.-S.M.); amalia-stefana-v-darie@d.umfiasi.ro (A.-S.T.); crisanadrian93@yahoo.com (A.C.); irina.costache@umfiasi.ro (I.-I.C.); ovidiu.mitu@umfiasi.ro (O.M.); 2 Department of Immunology, Faculty of Medicine, University of Medicine and Pharmacy “Gr. T. Popa”, 700115 Iasi, Romania; dconstantinescu_ro@yahoo.com (D.C.); ccianga@hotmail.com (C.M.C.); mariana.pavel86@gmail.com (M.P.-T.); 3 Department of Mother and Child Medicine, Faculty of Medicine, University of Medicine and Pharmacy “Gr. T. Popa”, 700115 Iasi, Romania; antondana66@yahoo.com; 4 Department of Infectious Diseases, Faculty of Medicine, University of Medicine and Pharmacy “Gr. T. Popa”, 700115 Iasi, Romania; larisa.miftode@yahoo.com; 5 Department of Morpho-Functional Sciences (II), Faculty of Medicine, University of Medicine and Pharmacy “Gr. T. Popa”, 700115 Iasi, Romania; ionela.serban@umfiasi.ro

**Keywords:** cardiac biomarkers, ST2, acute heart failure, prognosis, natriuretic peptides

## Abstract

Background: Acute heart failure (HF) represents an increasingly common and challenging presentation in the emergency room, also inducing a great socio-economic burden. Extensive research was conducted toward finding an ideal biomarker of acute HF, both in terms of sensitivity and specificity, but today practicians’ interest has shifted towards a more realistic multimarker approach. Natriuretic peptides (NPs) currently represent the *gold standard* for diagnosing HF in routine clinical practice, but novel molecules, such as sST2, emerge as potentially useful biomarkers, providing additional diagnostic and prognostic value. Methods: We conducted a prospective, single-center study that included 120 patients with acute HF and 53 controls with chronic HF. Of these, 13 patients (eight with acute HF, five from the control group) associated the coronavirus-19 disease (COVID-19). The diagnosis of HF was confirmed by a complete clinical, biological and echocardiographic approach. Results: The serum levels of all studied biomarkers (sST2, NT-proBNP, cardiac troponin) were significantly higher in the group with acute HF. By area under the curve (AUC) analysis, we noticed that NT-proBNP (AUC: 0.976) still had the best diagnostic performance, closely followed by sST2 (AUC: 0.889). However, sST2 was a significantly better predictor of fatal events, showing positive correlations for both in-hospital and at 1-month mortality rates. Moreover, sST2 was also associated with other markers of poor prognosis, such as the use of inotropes or high lactate levels, but not with left ventricle ejection fraction, age, body mass index or mean arterial pressure. sST2 levels were higher in patients with a positive history of COVID-19 as compared with non-COVID-19 patients, but the differences were statistically significant only within the control group. Bivariate regression showed a positive and linear relationship between NT-proBNP and sST2 (*r*(120) = 0.20, *p* < 0.002). Conclusions: we consider that sST2 has certain qualities worth integrating in a future multimarker test kit alongside traditional biomarkers, as it provides similar diagnostic value as NT-proBNP, but is emerging as a more valuable prognostic factor, with a better predictive value of fatal events in patients with acute HF.

## 1. Introduction

Acute heart failure (HF) represents the most severe display of the pathophysiological continuum that alters normal heart functioning, with a clinical presentation dominated by severe and rapidly progressive signs and symptoms. Acute HF, either de novo or as a decompensation of a previously diagnosed chronic HF, is one of the most common causes of hospitalization in developed countries, being characterized by unacceptably high mortality rates, frequent readmissions and significant socio-economic burden [1,2].

As a polymorphic syndrome, acute HF may present as a constellation of clinical phenotypes, such as acute pulmonary edema, cardiogenic shock or hypertensive HF [3]. Consequently, early identification, selection and hospitalization of patients with acute HF become of paramount importance for an appropriate therapeutic approach. Several biomarkers have been studied so far, but only the natriuretic peptides (NPs) have been routinely implemented in clinical practice. Traditionally, the B-type natriuretic peptide (BNP) and its amino-terminal prohormone (NT-proBNP) were extensively analyzed in multiple studies that included dyspneic patients with acute HF, their serum levels being correlated with symptoms’ severity, as measured by New York Heart Association (NYHA) functional class [4,5]. However, despite presenting high sensitivities and negative predictive values for the diagnosis of acute HF, the specificity of NPs is rather low, their concentrations being also influenced by several other cardiac and non-cardiac conditions, such as acute coronary syndromes, myocarditis, cardioversion, age, anemia, obesity and renal failure [6,7].

Therefore, additional imaging techniques or the use of improved biomarkers are required for a diagnostic certainty, as the evaluation of NPs alone is more useful in ruling out HF rather than establishing it, especially in cases without clinical aspects suggestive of HF. In this regard, echocardiography can detect an impaired left ventricular (LV) systolic function, but this may not necessarily represent the etiology of dyspnea since an important share of the population with reduced LV ejection fraction (LVEF) is asymptomatic. Similarly, dyspnea can coexist with a quasi-normal systolic function in patients with underlying pulmonary comorbidities and concomitant HF with preserved or mildly reduced LVEF [6,8].

Under these circumstances, we can outline the profile of an ideal biomarker: a high degree of sensitivity, specificity and reproducibility, reasonable cost and a simple assessment method—these aspects being required to initiate a prompt diagnostic and therapeutic approach. Such an alternative may be represented by sST2, which is the soluble isoform of the interleukin-1 receptor family member ST2. Several studies have highlighted the potential use of sST2 in patients with acute HF, based on its enhanced release by cardiac fibroblasts and cardiomyocytes in response to myocardial stretch. Moreover, sST2 may be a reliable marker of fibrosis, its release being directly related to some fibrogenetic conditions commonly found in HF, such as biomechanical strain and elevated Angiotensin II [9,10].

In order to emphasize the potential importance of serum sST2 assessment in patient with acute HF, we need to describe some morphofunctional particularities of this molecule. ST2 protein is expressed either as a soluble isoform (sST2) or a transmembrane receptor (ST2L); the ligand for both isoforms is represented by interleukin-33 (IL-33), but only by binding ST2L the IL-33 activates a cardioprotective signalling axis. However, under myocardial stress conditions (e.g., mechanical strain due to volume or pressure overload), sST2 is excessively released by cardiac fibroblasts and will competitively bind the IL-33, thus preventing it to attach the ST2L and subsequently inhibiting the cardioprotective effects [11,12].

Multiple literature data already confirmed that sST2 is presenting a high prognostic value in patients with acute HF, strongly predicting rehospitalizations and mortality rates, either alone or acting synergistically with NPs [10,12]. A very recent study even emphasized the superiority of sST2 compared with both NPs and cardiac troponins in predicting in-hospital fatal events [13].

Considering all these aspects and the increasing focus on the vast area of cardiac biomarkers, the goal of this research was to investigate the circulating levels of sST2 in patients with acute HF and the relationship between sST2 and other relevant parameters, thus aiming to potentially include this relatively new biomarker in a multimarker panel for an improved approach of acute HF in emergency room.

## 2. Materials and Methods

### 2.1. Study Design and Population

We conducted a prospective case-control study that evaluated consecutively enrolled patients with acute or chronic decompensated HF admitted in Cardiology Clinic of the *St. Spiridon* Emergency County Hospital (Iasi, Romania) between January 2021 and June 2021. The control group consisted of ambulatory patients, with stable, compensated HF. The diagnosis of acute HF was established in patients who presented in emergency with acute-onset dyspnea and/or other aspects suggestive for HF, as defined by Framingham criteria- consisting of major (paroxysmal nocturnal dyspnea, orthopnea, jugular venous distension, S3 gallop, cardiothoracic ratio > 0.5 on X-ray, aspect of pulmonary edema on X-ray, and pulmonary crackling rales) and minor (peripheral edema, nocturnal paroxysmal dyspnea, exertional dyspnea, hepatomegaly, pleural effusion and heart rate ≥100 bpm) criteria. The presence of two major or one major + two minor criteria is required to fulfill the criteria for HF. In all patients, the diagnosis of HF was further ascertained by echocardiography; an EF < 50% was considered abnormal and defined the so-called HF with reduced EF (HFrEF), while an EF > 50% doubled by diastolic dysfunction and/or increased LV filling pressures defines the HF with preserved EF (HFpEF).

The exclusion criteria were the inability to fully perform an adequate physical and/or echocardiographic examination (e.g., recent thoracic surgery, extreme obesity, severe thoracic malformations or hyperesthesia), the presence of late-stage cancer, severe ongoing infections, recent major surgery, untreated neuropsychiatric disorders or a value of NT-proBNP < 300 pg/mL. All patients aged < 18 years were also excluded.

The patients’ medical histories were obtained by performing detailed anamneses and from patients’ personal files or the hospital’s archives. Certain sociodemographic aspects (including age, gender, place of residence), particular behavioral conditions (tobacco use, toxic exposure, alcohol abuse-defined as >2 daily standard drinks for men and >1 for women), underlying diseases or current medication were thoroughly assessed. The associated pathologies (e.g., infectious diseases, pre-existing cardiovascular pathologies, diabetes mellitus) were either previously documented or diagnosed during current hospitalization. For statistical purposes we used only anthropometric indices (weight, height, body mass index) and office blood pressure values that were measured at admission. Diabetes mellitus was defined as a fasting blood glucose level ≥ 126 mg/dL, a HbA1c ≥ 6.5%, or current antidiabetic medication. Obesity was established in all patients presenting a body mass index (BMI) ≥ 30 kg/m^2^, while overweight was defined by a BMI between 25 and 29.9 kg/m^2^. At admission, all patients were screened for severe acute respiratory syndrome coronavirus type 2 (SARS-CoV-2), using polymerase chain reaction available tests.

A fasting venous blood sample was collected after admission, then centrifuged in order to separate the plasma/serum. Subsequently, the resulted samples were either immediately tested for routine investigations, or stored at −80 °C for up to six months, until the final analysis of the sST2. The sST2 was assessed using the enzyme-linked immunosorbent assay (ELISA) based kits (Abcam, Cambridge, UK). Dilutions were performed in order to adequately assess the large spectrum of concentrations met in our study group relative to the sensibility level of the commercial kit.

The echocardiograms were performed using a GE Vivid^TM^ V7 ultrasound device (General Electric, Boston, CA, USA).

The following cut-off values were used for defining the normal range: ST2 < 35 ng/mL, NT-proBNP < 300 pg/mL, high-sensitive cardiac troponin < 14 ng/L.

The study protocol was approved by the Ethics Committee of the Grigore T. Popa University of Medicine and Pharmacy and by the Ethics Committee of the Emergency Clinical Hospital *St. Spiridon.* All research was conducted according to the ethical guidelines of the Declaration of Helsinki Principles, revised in 2013. All patients have signed a standard written informed consent in order to participate in this study.

### 2.2. Statistical Analysis

We used Kolmogorov–Smirnov test for the assessment of the normal distribution of continuous variables in the study population. Normally distributed parameters are presented as medians plus standard deviation; to compare the mean values (in the case of continuous variables) we used the Student’s t-test and one-way ANOVA. Concerning those variables not normally distributed, to characterize the dispersion of the patients we used the median, the upper quartile (75th percentile correspondent) and the lower quartile (25th percentile correspondent), respectively, between which 50% of the values are included. Categorical variables were presented as frequencies and percentages. Differences between the acute HF group and the controls were assessed using parametric (independent sample *t*-test) or non-parametric (Mann–Whitney U) tests, as appropriate. The chosen level of statistical significance was 5% (*p* < 0.05).

The assessment of the correlation between two variables was performed using the correlation coefficients (r) Pearson and Spearman. The Pearson test assumes that both variables are continuous, while the Spearman test represents its non-parametric equivalent. The level of significance was the same—5%. Furtherly, we used linear regression to evaluate and illustrate the linear relationship between two correlated variables. It has predictive value by calculating an equation: y = ax + b (where y is the variable considered dependent and x is the independent variable), which allows the estimation of the behavior of the independent variable when the dependent variable is known. The regression coefficient is represented by the slope of the regression line. Moreover, to test the independent association between ST2 serum levels and both the traditional biomarkers (NT-proBNP, troponin), we performed a standard multiple regression, where ST2 was the dependent variable, while NT-proBNP and troponin were the predictors.

The diagnostic performance of the biomarkers in acute HF was evaluated by receiver operating characteristic (ROC) analysis, with the subsequent comparison of the areas under the curve (AUC). The cut-off values for ST2 were also drawn from the ROC curve, using Youden’s index or the point where sensitivity and specificity are equal (Se = Sp), as appropriate. The correlation between biomarkers concentration and survival rates was evaluated using Mantel–Cox (log-rank) test.

Data analysis was performed using IBM SPSS Statistics for Windows (version 23) and Microsoft Excel for organizing data before statistical processing. All tests were two-tailed and a *p*-value < 0.05 was considered statistically significant.

## 3. Results

### 3.1. Baseline Characteristics

The study included a total of 173 patients, divided in two groups: 120 patients diagnosed with acute decompensated HF and 53 patients with chronic stable HF, representing the control group. Overall mortality rate was 12.1%, all the 21 fatalities being recorded in patients with acute HF.

In Table 1 we highlighted some relevant demographic, clinical and biological characteristics of the included patients. Additionally, after performing comparisons between the baseline characteristics of the patients with acute HF and those from control group, we found a significantly higher prevalence of important cardiovascular risk factors among patients with acute HF, such as age, obesity, alcohol abuse and a low level of HDL-cholesterol. Moreover, the presence of ischemic heart disease was more common amongst patients with acute HF, as were other relevant parameters, such as impaired LVEF, electrolyte imbalances or elevated serum creatinine. Regarding other traditional cardiovascular risk factors, there were no significant differences between the two groups concerning the gender, smoking status or the presence of hypertension or diabetes mellitus. Being a study conducted during COVID-19 pandemic, 13 patients (8 with acute HF, 5 in control group) were associated with the coronavirus infection, either previously diagnosed or positively tested during hospitalization. With regard to medical therapy, the administration of loop-diuretics and mineralocorticoid receptor antagonists was significantly more common among patients with acute HF (*p* < 0.01), while beta-blockers and inhibitors of the renin–angiotensin–aldosterone system were more prescribed in control patients, as part of the standard therapy in clinically stable HF.

### 3.2. Profile of Biomarkers

As depicted in Table 2, the median values of sST2, troponin and NT-proBNP were all significantly higher in patients with acute HF compared with controls (*p* < 0.001). Consequently, the next step was to perform comparisons with various relevant clinical, echocardiographic or biological data, thus aiming to assess the potential correlations between these parameters and the analyzed biomarkers (Table 3).

All biomarkers exhibited modest positive direct correlations with each other, in the case of ST2 reaching the statistical significance when compared with both NT-proBNP (*p* = 0.014) and troponin (*p* = 0.019) (Table 4).

We noticed that sST2 serum levels did not show statistically significant differences between patients with HFrEF and those with HFpEF; a similar pattern was also valid in the case of NT-proBNP or troponin. All three analyzed biomarkers had significant positive and direct correlations with each other. Moreover, sST2 presented direct and highly significant correlations with some clinical manifestations typical for acute HF, such as pulmonary crackles and peripheral edema. We also noticed a linear association between high levels of sST2 and elevated serum lactate or increased need for inotropic support, which represent well-established poor prognosis factors in HF. Compared with NT-proBNP, sST2 was a better predictor of fatal events, with positive correlation for both in-hospital and 1-month mortality rates. No significant association was found between sST2 and age, BMI or inflammation (expressed as C-reactive protein).

In an acute setting of HF, in order to avoid further unnecessary and time-consuming explorations, it is critical to have a biomarker able to ascertain the cardiac etiology of the symptoms, whether the source is right, left or global HF. Thus, we compared the levels of sST2, NT-proBNP and troponin in patients with isolated right ventricle dysfunction (12 cases) to those presenting with LV dysfunction or even with global HF (108 cases), with detailed data provided in Table 5. Unlike troponin, sST2 and NT-proBNP showed no significant variations depending on the type of HF, confirming their utility in confirming both right and left ventricular dysfunction, especially in cases with an equivocal clinical presentation. Focusing on sST2, we observed that its serum levels in the two subgroups were very similar (140.9 ± 119.5 versus 123.8 ± 135.1, *p* = 0.64).

### 3.3. Diagnostic Performance of Biomarkers

**Figure 1:**
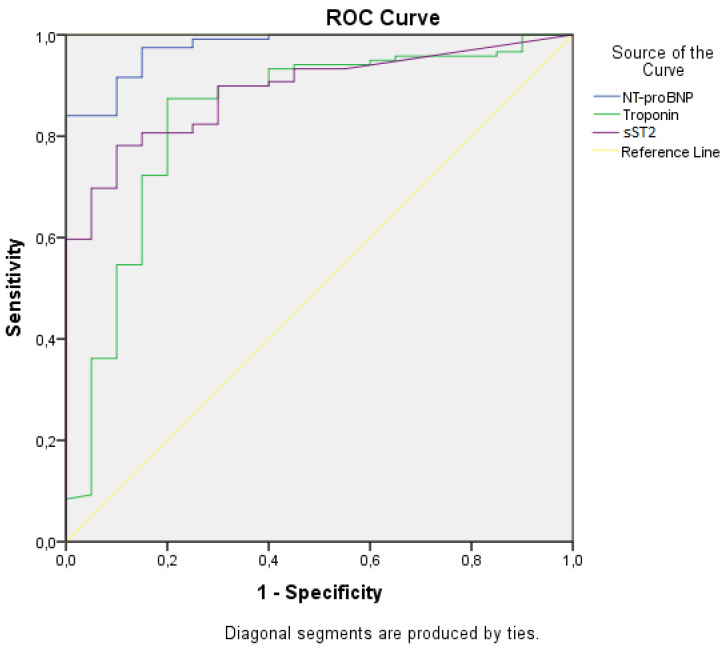
Receiver operating characteristic (ROC) curves for specified biomarkers.

The assessment of diagnostic performance is an essential aspect when it comes to biomarkers used in life-threatening pathologies, such as acute HF. ROC analysis revealed that the curves show an adequate diagnostic performance for all three biomarkers (Figure 1), with the AUCs for sST2, NT-proBNP and troponin being all higher than 0.8 and statistically significant (*p* < 0.05) in diagnosing acute HF (Table 6). With an AUC of 0.889, sST2 exhibited significant potential in diagnosing acute HF, very similar to the well-established NT-proBNP (AUC = 0.976). Although with a lower diagnostic performance (AUC = 0.838), cardiac troponin continues to be an important biomarker, butit is rather addressed to the diagnosis of HF due to acute myocardial ischemia.

Further, we aimed to drawn from the ROC curve the optimal cut-off value for ST2, which can adequately identify patients at risk. By using two validated methods, we found that a cut-off value of 29 ng/mL has 80.7% sensitivity and 80.8% specificity in diagnosing acute HF, while a 36 ng/mL cut-off (very similar to the internationally accepted cut-off value of 35 ng/mL) had a significantly improved specificity of 90.1% at the expense of only a slight decrease in sensitivity (78.2%) (Table 7). It is worth mentioning that exceeding any of these cut-offs was not associated with significantly increased mortality. However, when considering the already validated “high-risk” cut-off value of 70 ng/mL, characterized by a 63.9% sensitivity and 95.2% specificity, we observed a significantly elevated risk of death among patients having ST2 serum levels above this threshold (OR 3.9 [95% CI 2.2–12.2], *p* = 0.01). Specifically for our study, we identified a lower concentration related to a “high-risk” profile, a 60 ng/mL cut-off (68.8% sensitivity and 92.9% specificity) being also significantly associated with increased mortality (OR 3.3 [95%CI 1.1–9.8, *p* = 0.04]).

**Figure 2:**
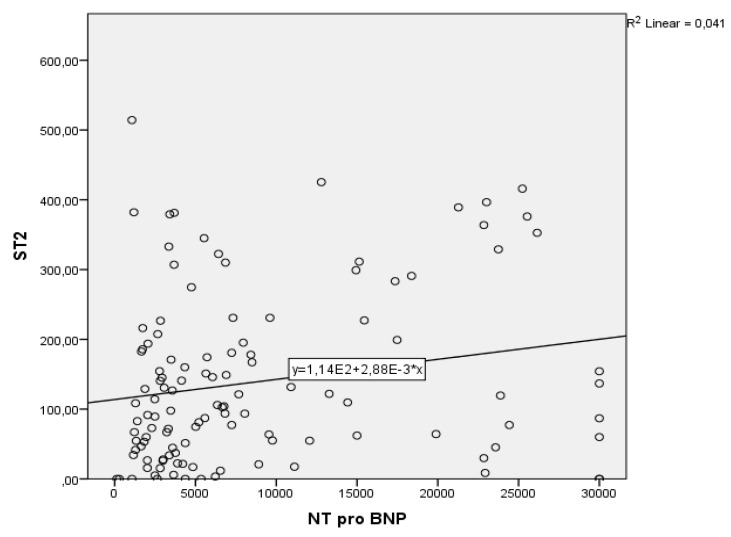
Correlation between ST2 and NT-proBNP.

The next step was to perform a bivariate regression to examine how the NT-proBNP levels can predict the sST2 levels. A scatterplot showed that the relationship between NT-proBNP levels and sST2 was positive and linear and did not reveal any bivariate outliers. The correlation between these two parameters was statistically significant, r(120) = 0.20, *p* < 0.002. The regression equation for predicting sST2 from NT-proBNP was y = 113.85 + 0.003x. The *r*^2^ for this equation was 0.041; that is, 4.1% of the variance in sST2 was predictable from the level of NT-proBNP. The bootstrapped 95% confidence interval for the slope to predict sST2 from NT-proBNP range from 0.001 to 0.005; thus, for each unit of increase in NT-proBNP, the sST2 increases with 0.003 (Figure 2).

**Figure 3:**
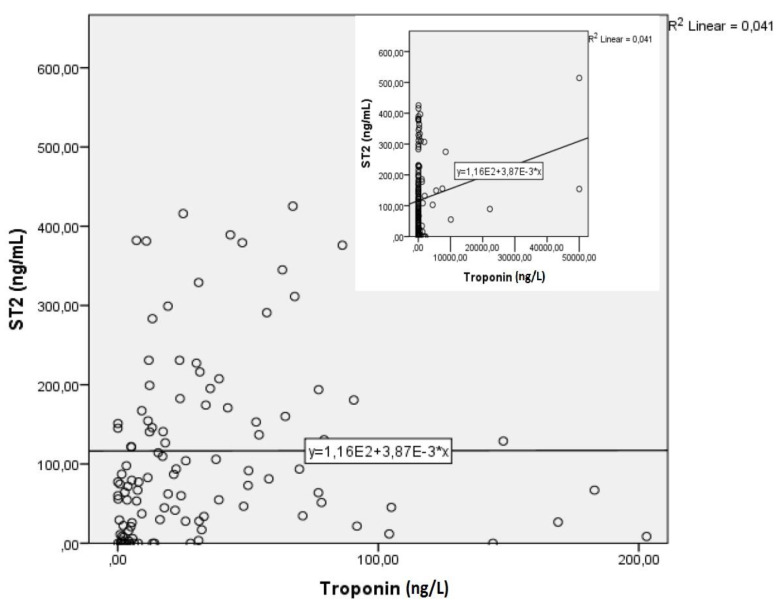
Correlation between ST2 and troponin.

Similarly, the correlation between sST2 and troponin was statistically significant, r(120) = 0.18, *p* < 0.003. The regression equation for predicting sST2 from troponin was y = 134.22 + 0.003x, while the *r*^2^ for this equation was 0.036. The bootstrapped 95% confidence interval for the slope to predict the sST2 from troponin range from 0.001 to 0.007; basically, for each unit of increase in troponin, the sST2 increases by 0.003 (Figure 3).

Further, a standard multiple regression was performed to assess the ability of NT-proBNP and troponin levels to predict the ST2 levels in our study population. Preliminary analyses were performed to ensure there was no violation of the assumption of normality, linearity and multicollinearity. A significant regression equation was found (F = 4.445, *p* = 0.01), with an *r*^2^ of 0.071 and adjusted r-square of 0.055. The predicted ST2′s value was equal to 110.99 + 0.003x (NT-proBNP or troponin). Basically, for every increase in one unit of NT-proBNP, ST2 levels increase by 0.003 and, similarly, for every increase in one unit of troponin, ST2 levels increase by 0.003. The bootstrapped 95% confidence interval for the slope to predict ST2 from NT-proBNP ranged from 0.001 to 0.005, while, for troponin, it ranged from 0.001 to 0.004. In conclusion, both NT-proBNP and troponin were significant predictors of ST2 concentrations in our multimarker model comprising patients with acute HF.

Finally, given the pandemic situation and the vastly incriminated role of COVID-19 in cardiovascular pathology, we considered it appropriate to assess the serum levels of sST2 in both groups (acute HF and controls), depending on their COVID-19 status. We found that sST2 was significantly increased in control group with COVID-19 compared with non-COVID control patients [79.3 ng/mL (IQR: 18.9–151.8) versus 3.3 ng/mL (IQR: 0.05–27.4), *p* < 0.01]. On the other hand, in patients with acute HF, even if we noticed a slightly increased sST2 among COVID-19 patients compared with non-COVID ones, the difference did not reach the threshold of statistical significance (112.3 ng/mL [IQR: 48.7–199.1] versus 107.2 ng/mL [46.3–196.9], *p* = 0.301).

## 4. Discussion

Despite the growing awareness among clinicians, researchers and even patients, HF still remains one of the main sources of morbidity, mortality and significant healthcare costs, in both developing and developed countries [14,15]. In the context of a globally rising incidence and prevalence of HF [16] and due to its polymorphic clinical presentation that lead to high rates of misdiagnosis, the need for rapid and accurate diagnostic tools is becoming of utmost importance. Therefore, cardiac biomarkers lately represented a fertile research area concerning the diagnostic approach of HF and, particularly, acute HF. Even if there is a plethora of new HF biomarkers [17], in our study we focused on sST2- a relatively novel biomarker with promising results- aiming to a head-to-head comparison with NT-proBNP and high-sensitive troponin, respectively.

When designing the study, we tried to reproduce a model similar to the routine clinical practice of an emergency hospital. In this regard, we enrolled patients with confirmed acute HF, while the control group comprised stable patients with chronic HF. We would like to stress that both groups were presenting various associated pathologies, such as diabetes, obesity, hypertension, ischemic heart disease or even COVID-19. These aspects may reflect not only the multifactorial etiology of HF, but may also represent additional triggers for acute HF and, therefore, possible therapeutic targets in order to avoid further decompensation episodes [1]. The results from our study confirmed the important etiopathogenic role of the comorbidities, as the patients with acute HF had presented a significantly higher incidence of ischemic heart disease and obesity. Moreover, the presence of a significantly reduced LVEF compared with control group basically reflects the pathophysiological continuum between obesity, atherosclerosis, myocardial ischemia and systolic dysfunction due to ischemic HF.

With regard to the levels of sST2, we noticed a significant elevation among patients with acute HF, compared with controls with chronic HF. Contrasting to other studies [10,17], we did not find a significant difference in sST2 levels between those with HFpEF and HFrEF. This may be explained by the fibrogenesis stimulus induced by the sST2 in the context of an increased LV filling pressures—patients with HFpEF are generally hypertensive and obese, with elevated Angiotensin II circulating levels, an aspect that is furtherly inducing myocardial hypertrophy, increased collagen synthesis and subsequent fibrosis [11]. On the other hand, in patients with HFrEF, the impaired systolic function is directly related to an increased myocardial strain, that will subsequently induce additional sST2 release, thus preventing the cardioprotective ST2L-IL-33 interaction and, ultimately, closing a vicious pathophysiological circle [18].

Diastolic dysfunction and left atrial (LA) enlargement are common echocardiographic features among patients with HF. Our results showed that sST2 is associated with increased LV filling pressures (expressed as E/e’), but not with an increased LA volume, a finding similar to those from other studies [19,20], but opposed to the results obtained by Najjar et al. who found a correlation between sST2 and LA indexed volume, but not with E/e’ [10]. However, LA strain—as a possible source for sST2 release—is independent of LA volume, as the LA fibrosis and the subsequent atrial cardiomyopathy are rather related to different pathophysiological pathways (e.g., inflammation, oxidative stress, electrical alterations), as shown in PARAMOUNT trial and other literature data [21,22]. We did not notice any association between sST2 and LVEF, a finding that is consistent with some previous literature data [10,23], but discordant to studies that highlighted an association between increased sST2 and reduced LVEF following an acute myocardial infarction [24] or in patients with acute onset dyspnea [25,26]. In this context, we observed that sST2 was also associated with increased need for inotropic agents, a relationship linked to a poor prognosis, as also previously suggested by Dolapoglu et al. [27].

Given sST2′s already documented quality in identifying myocardial dysfunction in patients with HFpEF [9,10,28], we aimed to assess sST2′s potential to be a so-called ’dual’ biomarker, for both right and left ventricular dysfunction. For this purpose, we compared a group of patients with acute HF due to isolated RV dysfunction to the rest of the patients, who presented only LV dysfunction or even a global HF (LV ± RV dysfunction). The results revealed indiscriminately increased levels of sST2 in both groups, without statistically significant differences. Basically, this confirms the utility of sST2 in detecting even isolated RV dysfunction, in the absence of echocardiographic aspects suggestive for LV systolic or diastolic dysfunction. Correspondingly, the involvement of sST2 in the functioning of the RV was also observed by Shah et al., who identified a correlation between high serum sST2 and an impaired RV systolic function expressed as diminished RV fractional area change [25], while another study highlighted the increased sST2 in patients with chronic thromboembolic or idiopathic pulmonary hypertension [29].

A very actual issue in clinical practice refers to the mutual interdependence between COVID-19 and HF; the viral infection acts as a trigger for the decompensation of a chronic HF, while a preexisting cardiovascular disease increases the risk for severe clinical manifestations during the infection [30,31]. COVID-19 may even cause a de novo HF, especially acute RV failure due to pulmonary embolism [32] or in the context of acute respiratory distress syndrome due to severe viral pneumonia. COVID-19 determines an increase in the pulmonary vascular resistance via multiple mechanisms: it enhances the release of vasocontrictive mediators, causes a hypoxia-mediated pulmonary vasoconstriction and promotes a hypercoagulable status that leads to capillary microthrombosis and even creates the conditions for extrinsic vascular compression due to interstitial edema or pleural effusion [11,33]. This wide range of incriminated mechanisms induces an important mechanical strain on the rather thin walls of the RV, thereby increasing sST2 release and accelerating fibrogenetic processes. Our results showed a significant increase in sST2 levels in control patients with COVID-19 compared with their non-infected counterparts, an aspect that may suggest an ongoing subclinical myocardial injury with a subsequent sST2 release. Concerning the patients with phenomena of acute HF, we observed that the serum concentrations of sST2 were higher among COVID-19 confirmed cases, but without reaching a statistical significance. However, due to the variable timespan between the initial diagnosis of COVID-19 and the actual admission for HF, doubled by the lack of repeated sST2 determinations, a clear conclusion cannot be draw concerning the dynamic change of sST2 levels in patients with HF and COVID-19.

Unlike NT-proBNP, in our study sST2 was significantly associated with several clinical aspects suggestive for congestive HF, such as pulmonary crackles and peripheral edema. The molecular substrate of this finding was recently explained by Pascual-Figal et al., who observed high concentrations of ST2 in bronchial aspirates of patients with cardiogenic pulmonary edema due to an increased myocardial strain. Very interesting, sST2 concentrations in bronchial aspirates presented direct correlations not only with serum levels of sST2, but also with NT-proBNP and cardiac troponin [34]. The same study also drew attention towards the extracardiac (especially pulmonary) sites of ST2 production, which are similarly activated by significant myocardial stress, such as acute HF after an induced acute myocardial infarction [34]. The increased secretion of sST2 was immunohistochemically detected in the epithelial alveolar pneumocytes of rats with ischemic HF, with a positive correlation between alveolar wall thickness and sST2 expression. This finding was further confirmed on cultures of type II human pneumocytes subjected to biomechanical strain, with the consequent induction of an increased secretion of sST2. Moreover, the same authors indicated leukocytes as possible sites for sST2 production, thus bridging the active role of IL-33 in inflammation to the systemic inflammatory status commonly found in HF [34,35]. The results from our study support the above-mentioned findings: patients with acute HF were presenting leukocytosis (>10000 cells/mm^3^) that was significantly associated with sST2 levels.

Similar to the results from the PRIDE study [26], we found that sST2 concentrations at admission correlated with NT-proBNP, creatinine clearance and lactate level, but not with age, gender or BMI. This may be regarded as an advantage of sST2 compared with NPs, whose values are allegedly influenced by these latter aspects in many studies [36,37,38], but not in ours. Although we observed an association between increased serum sST2 and elevated creatinine, the crude levels of sST2 are not directly influenced by the renal function, as opposed to NPs, where a significant decrease in their urinary excretion contributes to a high serum concentration [39]. The relative independence of sST2 of renal function is based on the rather small size of the ST2 molecule of ~50 kDa, which is very similar to the molecular weight cut-off for glomerular filtration (20–50 kDa) [40,41]. Thereby, a reasonable assumption is to consider that elevated sST2 levels in patients with acute HF are not caused by decreased renal clearance, but are rather a consequence of increased cardiac or pulmonary secretion, as previously stated [34]

Concerning the association between ST2, NT-proBNP and troponin, we observed direct (albeit modest) correlations between the three analyzed biomarkers, suggesting that, to a certain point, these biomarkers assess different pathophysiological mechanisms involved in HF syndrome. The cross-influence between ST2 and NT-proBNP (expressed as a significant direct correlation) can be partly explained by the fact that both biomarkers are sensitive to myocardial stretch, exhibiting dynamic serum level fluctuations and greatly depending on the ventricular load or the effectiveness of the administered therapy (e.g., loop-diuretics) [42]. The differences lie in sST2′s ability to reflect a plethora of HF mechanisms, such as fibrosis, myocardial inflammation or collagen deposition with subsequent cardiac remodeling, aspects that finally lead to impaired ventricular geometry and symptomatic HF [42,43]. Furthermore, despite the common final pathway representing the expression of myocardial strain, differences may be induced by the multiple confounding factors that are traditionally cited in literature for erratically altering NT-proBNP’s levels, such as age, obesity or renal dysfunction [12,34,44].

The important prognostic role of sST2 has already been established in several previous studies [10,12,27,29,45] and our results just further ascertained that sST2 is an important predictor of mortality, both in-hospital and at 1-month follow-up. Beside the fact that sST2 was significantly associated with short-term adverse outcomes in patients with acute HF enrolled in our study, we can also consider it as a useful tool for patients’ follow-up, whether they are presenting stable or decompensated HF.

A promising scenario assumes a dynamic assessment of sST2: starting with a baseline value at admission, then followed by seriated measurements during hospitalization in order to initiate additional drugs or to augment the doses of the preexisting ones [12]. One study showed that patients with persistently elevated values of sST2 in whom the beta-blockers were titrated to high doses presented a more favorable outcome as compared with those maintained on low-to-medium doses [46]. The central pillar of these dynamic measurements is represented by the internationally recognized sST2 cut-off value of >35 ng/mL, which was associated with worse prognosis in patients with HF [47]. Moreover, some authors observed that the period of time spent with sST2 above the cut-off level is associated with poor outcome and high mortality rates, whereas a rapid decrease below the cut-off point was suggestive for a better survival rate [48,49]. In our study, the median sST2 concentration in patients with acute HF (107.2 ng/mL) was well above the generally accepted cut-off value, and was associated with increased severity of symptoms and the need for immediate hospitalization and therapeutic approach. This finding is in line with the relatively new concept of a ’high-risk’ cut-off of >70 ng/mL, which was proposed to better distinguish dyspeic patients with high risk of acute HF. In these patients, the admission to the cardiology ward and the initiation of aggressive medications, such as loop diuretics and different antiremodeling drugs, are highly recommended [50]. In our study, the classical cut-off of 35 ng/mL presented good sensitivity and specificity in diagnosing acute HF but was not associated with a worse short-term outcome. Switching to the more specific but less sensitive 70 ng/mL cut-off, the predictive value of ST2 greatly improved, the patients with serum levels above this threshold having a four-fold increase in the risk of mortality, compared with those whose ST2 was below 70 ng/mL. Given that the cut-off value of 35 ng/mL in predicting adverse events is based on long periods of follow-up and serial measurements, our results and multiple evidence from literature [49,50,51] suggest that, in patients with suspected acute HF, a cut-off value of 70 ng/mL may be more useful in predicting short-term negative outcome.

With regard to our findings, it is important to highlight that the majority of the above-mentioned studies underlined the important prognostic value of sST2, that was cumulative or even superior to that of NT-proBNP. Given the certain particularities of each biomarker, their different pathophysiologic pathways, expression or even clearance, we consider that the development of a multimarker test kit comprising sST2 and the classical biomarkers will provide incremental diagnosis and prognosis information concerning patients with acute HF.

## 5. Conclusions

We focused our research on depicting the potential use of sST2 in clinical practice as a diagnostic and prognostic biomarker in patients presenting with phenomena suggestive of HF. In this regard, our results demonstrated the strong diagnosis value exhibited by sST2 in patients with acute HF, very similar to the *gold-standard* NT-proBNP. Moreover, it even presented a superior prognosis value compared with NT-proBNP, an increased serum sST2 at admission being significantly associated with a negative outcome and high mortality rates.

The sST2′s potential use in emergency room is further based on its preserved ability to confirm the diagnosis of HF whether it is caused by a right, left, or biventricular dysfunction, and regardless of LVEF. We also noticed that sST2, as opposed to NT-proBNP, is not influenced by certain confounding parameters that could alter its serum levels, such as non-modifiable constitutional aspects (e.g., age, gender), or commonly found conditions in patients with HF (e.g., obesity, renal dysfunction).

These aspects turn the spotlight on a new paradigm: the increasing interest in using a multimarker approach in patients with acute HF. In addition to the classical, routinely used biomarkers, the additive value provided by the novel biomarkers, namely sST2, may significantly enhance the diagnosis and prognosis accuracy in HF, thus leading to a more adequate therapeutic approach and a better risk stratification of these patients. Of course, performing dynamic assessments of sST2 would certainly represent a more valuable diagnosis and prognosis tool, but also a limitation in the wide-scale use of this emerging biomarker. Nevertheless, given the various pathophysiological mechanisms expressed at the myocardial level even in a subclinical manner, we consider that a scenario of associating multiple biomarkers in a standardized test kit might be a realistic future direction in the approach of patients with HF.

## 6. Limitations

The relatively small sample size and the unicentric design of the study were the most important limitations. Additionally, the biomarkers were measured only at admission, as only a single sST2 ELISA kit was available for each patient. A dynamic assessment by performing repeated measurements could potentially reflect the progression of HF, hence improving the prognosis value of sST2. However, it must be taken into account that the study was conducted in a pandemic period, with difficult enrollment procedures and limited options for the on-site follow-up visits and subsequent blood-sampling of the discharged patients.

## Figures and Tables

**Table 1:** Baseline characteristics of patients with acute HF and control group.

Characteristics	Total (*n* = 173)			Acute HF (*n* = 120)			Control Group (*n* = 53)			*p*-Value
	Min	Mean ± STD	Max	Min	Mean ± STD	Max	Min	Mean ± STD	Max	
**Age (years)**	18	65 ± 13.3	94	18	66.4 ± 15.3	94	30	64 ± 11.9	85	0.047
**Mortality rate**		21 (12.1%)			21 (17.5%)			0 (0%)		<0.001
**Gender**										0.438
Male: N, (%)		104 (60%)			71(59.20%)			33(62.30%)		
Female: N, (%)		69 (40%)			49(40.80%)			20(37.70%)		
**Area of residence**										0.353
Urban: N, (%)		Urban: 92 (53.2%)			Urban: 61 (50.8%)			Urban: 31 (58.5%)		
Rural: N, (%)		Rural: 81 (46.8%)			Rural: 59 (49.2%)			Rural: 22 (41.5%)		
**Smokers: N, (%)**		67 (38.7%)			48 (40%)			19 (35.8%)		0.605
**Alcohol abuse: N, (%)**		97 (56.1%)			75 (62.5%)			22 (41.5%)		0.012
**Hypertension**		94 (54.3%)			60 (50%)			34 (64.2%)		0.085
**Ischemic heart disease**		76 (43.9%)			59 (49.2%)			17 (32%)		0.037
**Diabetes mellitus**		29 (16.8%)			22 (18.3%)			7 (13.2%)		0.406
**Obesity (BMI > 30 kg/m^2^)**		49 (28.3%)			42 (35%)			7 (13.2%)		0.003
**COVID-19**		13 (7.5%)			8 (6.7%)			5 (9.4%)		0.241
**LV ejection fraction (%)**	10%	39.4 ± 14.4	72%	10%	33.8 ± 13.9%	61%	38%	52.2 ± 15.7	72%	0.017
**Hemoglobin (g/dL)**	7.20	13.27 ± 2.1	33.70	7.20	13.18 ± 2.4	33.70	9.60	13.48 ± 1.9	18.40	0.069
**Hematocrit (%)**	22.20	39.50 ± 9.7	56.30	22.20	39.54 ± 8.9	56.30	29.22	39.39 ± 11.2	52.70	0.909
**Leukocytes (×10^9^/L)**	1.20	9.52 ± 1.27	30.18	4.10	10.22 ± 1.34	25.20	1.19	7.93 ± 1.21	30.18	0.044
**Platelets (×10^3^/μL)**	37	266 ± 43	2630	37	270 ± 45	2630	37	253 ± 42	595	0.245
**Blood glucose (mg/dL)**	63	142.56 ± 37.1	582	63	149.21 ± 33.4	582	73	128.13 ± 41.8	388	0.111
**Total bilirubin (mg/dL)**	0.09	0.98 ± 0.19	5.02	0.10	1.18 ± 0.21	5.02	0.09	0.54 ± 0.18	1.12	<0.001
**Sodium (mmol/L)**	121	138.26 ± 12.6	147	121	137.77 ± 14.1	147	122	141.13 ± 8.5	147	0.002
**Potassium (mmol/L)**	2.90	4.50 ± 0.83	6.30	2.90	4.61 ± 0.88	6.30	3.20	4.27 ± 0.74	5.60	0.009
**Creatinine (mg/dL)**	0.60	1.15 ± 0.29	4.01	0.60	1.22 ± 0.31	4.01	0.68	0.99 ± 0.24	3.78	0.029
**Total cholesterol (mg/dL)**	63	164.15 ± 51.2	331	63	161.10 ± 51.9	331	90	173.28 ± 49.3	285	0.109
**LDL-cholesterol (mg/dL)**	33	109.23 ± 33.4	255	33	106.62 ± 35.6	255	53	124.60 ± 31.2	197	0.253
**HDL-cholesterol (mg/dL)**	12	41.68 ± 16.5	111	12	39.98 ± 15.9	111	36	52.35 ± 17.7	76	<0.001
**ACEI/ARBs/ARNi**		121 (70%)			77 (64.2%)			44 (83.1%)		0.012
**Beta-blockers**		148 (85.6%)			99 (82.5%)			49 (92.5%)		0.087
**MRA**		92 (53.2%)			83 (69.2%)			9 (17%)		<0.001
**Loop-diuretics**		119 (68.8%)			102 (85%)			17 (32.1%)		<0.001

N—number, STD—standard deviation, LV—left ventricle, BMI—body mass index, COVID-19—coronavirus disease 2019, LDL—low density lipoprotein, HDL—high density lipoprotein, ACEI—angiotensin-converting enzyme inhibitors, ARBs—angiotensin II receptor blockers, ARNi—angiotensin receptor-neprilysin inhibitors, MRA—mineralocorticoid receptor antagonist.

**Table 2:** Biomarker profile in patients with acute HF as compared withwith the control group.

Biomarker	Total (*n* = 173)	Acute HF (*n* = 120)	Control Group (*n* = 53)	*p*-Value
sST2 (ng/mL)	81.26 (24.20–172.60)	107.20 (45.61–194.89)	29.27 (3.10–107.06)	<0.001
NT-proBNP (pg/mL)	3757 (1827–9764)	5440 (2812–12791)	107.80 (41.30–325.25)	<0.001
Troponin (ng/L)	31.01 (7.03–104.80)	38.25 (12.45–179.50)	2.26 (1.14–5.43)	<0.001

sST2—soluble ST2, NT-proBNP—amino-terminal pro-brain natriuretic peptide. Values are expressed as medians (IQR—interquartile range).

**Table 3:** Correlations between biomarker levels and clinical, echocardiographic and biological variables in patients with acute HF.

Parameters	sST2		NT-proBNP		Troponin	
	*p*	r	*p*	r	*p*	r
LVEF	0.85	−0.01	0.89	−0.13	0.12	−0.14
LAVI	0.66	−0.04	0.72	0.03	0.38	−0.08
E/e’	0.05	0.23	0.06	0.21	0.22	0.18
C-reactive protein	0.421	0.07	0.01	0.23	0.919	0.01
Hemoglobin	0.728	−0.032	0.107	0.148	0.182	0.123
Leukocytes	<0.001	0.317	−0.131	0.152	<0.001	0.36
Creatinine	0.03	0.381	0.531	0.06	0.076	0.173
BMI	0.918	0.003	0.781	−0.03	0.907	0.03
Age	0.065	0.48	0.221	−0.113	0.945	0.006
MAP	0.40	−0.07	0.09	−0.15	0.06	−0.17
Pulmonary crackles	0.01	0.29	0.191	0.120	0.42	−0.072
Peripheral edema	0.01	0.22	0.719	0.03	0.320	−0.091
Lactate level	<0.001	0.49	0.296	0.09	<0.001	0.31
Inotropic support	<0.001	0,34	0.1	0.105	0.214	0.114
In-hospital mortality	<0.001	0.40	0.269	0.102	0.02	0.21
1-month mortality	<0.001	0.42	0.371	0.082	0.03	0.13

LVEF—left ventricular ejection fraction, LAVI—left atrium indexed volume, E/e’—left ventricular transmittal early diastolic filling velocity/left ventricular early diastolic myocardial velocity, BMI—body mass index, MAP—mean arterial pressure.

**Table 4:** Correlation analysis concerning levels of biomarkers.

		ST2	NT proBNP	Troponin
Pearson Correlation	ST2	1.000	0.202	0.189
	NT proBNP	0.202	1000	0.081
	Troponin	0.189	0.081	1.000
Sig. (1-tailed)	ST2	.	0.014	0.019
	NT proBNP	0.014	.	0.190
	Troponin	0.019	0.190	.

**Table 5:** The variability of biomarkers’ serum level in in patients with isolated right RV dysfunction compared with those with LV± RV dysfunction.

BIOMARKER	HF Phenotype	N	Mean	Standard Deviation	Standard Error Mean	*p* Value
**ST2** (ng/mL)	LV ± RV dysfunction	108	140.9	119.5	11.5	
	isolated RV dysfunction	12	123.8	135.1	39.0	0.64
**NT-proBNP** (pg/mL)	LV ± RV dysfunction	108	9043.5	8476.4	815.6	
	isolated RV dysfunction	12	6866.1	8333.2	2405.5	0.40
**Troponin** (ng/L)	LV ± RV dysfunction	108	1648.1	7172.6	690.1	
	isolated RV dysfunction	12	193.2	317.1	91.5	0.02

LV—left ventricle, RV—right ventricle, HF—heart failure.

**Table 6:** Diagnostic test performance for cardiac biomarkers in acute HF.

Biomarker	AUC	Standard Error	95% Confidence Interval		*p* Value
			Lower Bound	Upper Bound	
NT-proBNP (pg/mL)	0.976	0.013	0.951	1.000	<0.05
sST2 (ng/mL)	0.889	0.031	0.829	0.949	<0.05
Troponin (ng/L)	0.838	0.055	0.731	0.946	<0.05

AUC—area under curve.

**Table 7:** Cut-off values for sST2.

Criterion	Cut-Off Value ST2 (ng/mL)	Se	Sp
Se = Sp	29	0.807	0.808
Maximum Youden’s index (Se + Sp)	36	0.782	0.901
“High-risk” cut-offs	70 60	0.639 0.688	0.952 0.929

## Data Availability

The data presented in this study are available within the article.

## References

[1] Farmakis D., Parissis J., Lekakis J., Filippatos G. (2015). Acute heart failure: Epidemiology, risk factors, and prevention. Rev. Esp. Cardiol. Engl. Ed..

[2] Čerlinskaitė K., Javanainen T., Cinotti R., Mebazaa A., Global Research on Acute Conditions Team (GREAT) Network (2018) (2018). Acute Heart Failure Management. Korean Circ. J..

[3] Inamdar A.A., Inamdar A.C. (2016). Heart Failure: Diagnosis, Management and Utilization. J. Clin. Med..

[4] Januzzi J.L., Camargo C.A., Anwaruddin S., Baggish A.L., Chen A.A., Krauser D.G., Tung R., Cameron R., Nagurney J.T., Chae C.U. (2005). The N-terminal Pro-BNP investigation of dyspnea in the emergency department (PRIDE) study. Am. J. Cardiol..

[5] Maisel A.S., Krishnaswamy P., Nowak R.M., McCord J., Hollander J.E., Duc P., Omland T., Storrow A.B., Abraham W.T., Wu A.H. (2002). Rapid measurement of B-type natriuretic peptide in the emergency diagnosis of heart failure. N. Engl. J. Med..

[6] Nadar S.K., Shaikh M.M. (2019). Biomarkers in Routine Heart Failure Clinical Care. Card. Fail. Rev..

[7] Roberts E., Ludman A.J., Dworzynski K., Al-Mohammad A., Cowie M.R., McMurray J.J., Mant J., NICE Guideline Development Group for Acute Heart Failure (2015). The diagnostic accuracy of the natriuretic peptides in heart failure: Systematic review and diagnostic meta-analysis in the acute care setting. BMJ.

[8] Obokata M., Olson T.P., Reddy Y.N.V., Melenovsky V., Kane G.C., Borlaug B.A. (2018). Haemodynamics, dyspnoea, and pulmonary reserve in heart failure with preserved ejection fraction. Eur. Heart J..

[9] Song Y., Li F., Xu Y., Liu Y., Wang Y., Han X., Fan Y., Cao J., Luo J., Sun A. (2020). Prognostic value of sST2 in patients with heart failure with reduced, mid-range and preserved ejection fraction. Int. J. Cardiol..

[10] Najjar E., Faxén U.L., Hage C., Donal E., Daubert J.-C., Linde C., Lund L.H. (2019). ST2 in heart failure with preserved and reduced ejection fraction. Scand. Cardiovasc. J..

[11] Miftode R.S., Petriș A.O., Onofrei Aursulesei V., Cianga C., Costache I.I., Mitu O., Miftode I.L., Șerban I.L. (2021). The Novel Perspectives Opened by ST2 in the Pandemic: A Review of Its Role in the Diagnosis and Prognosis of Patients with Heart Failure and COVID-19. Diagnostics.

[12] Villacorta H., Maisel A.S. (2016). Soluble st2 testing: A promising biomarker in the management of heart failure. Arq. Bras. Cardiol..

[13] Borovac J., Glavas D., Grabovac Z.S., Domic D.S., Stanisic L., D’Amario D., Kwok C.S., Božić J. (2020). Circulating sST2 and catestatin levels in patients with acute worsening of heart failure: A report from the CATSTAT-HF study. ESC Heart Fail..

[14] Davari M., Maracy M.R., Khorasani E. (2019). Socioeconomic status, cardiac risk factors, and cardiovascular disease: A novel approach to determination of this association. ARYA Atheroscler..

[15] Camps-Vilaró A., Delgado-Jiménez J.F., Farré N., Tizón-Marcos H., Álvarez-García J., Cinca J., Dégano I.R., Marrugat J. (2020). Estimated Population Prevalence of Heart Failure with Reduced Ejection Fraction in Spain, According to DAPA-HF Study Criteria. J. Clin. Med..

[16] Chioncel O., Lainscak M., Seferovic P.M., Anker S., Crespo-Leiro M.G., Harjola V.P., Parissis J., Laroche C., Piepoli M., Fonseca C. (2017). Epidemiology and one-year outcomes in patients with chronic heart failure and preserved, mid-range and reduced ejection fraction: An analysis of the ESC Heart Failure Long-Term Registry. Eur. J. Hear. Fail..

[17] Jirak P., Pistulli R., Lichtenauer M., Wernly B., Paar V., Motloch L.J., Rezar R., Jung C., Hoppe U.C., Schulze P.C. (2020). Expression of the Novel Cardiac Biomarkers sST2, GDF-15, suPAR, and H-FABP in HFpEF Patients Compared with ICM, DCM, and Controls. J. Clin. Med..

[18] Sanada S., Hakuno D., Higgins L.J., Schreiter E.R., McKenzie A.N.J., Lee R.T. (2007). IL-33 and ST2 comprise a critical biomechanically induced and cardioprotective signaling system. J. Clin. Investig..

[19] Shah K.B., Kop W.J., Christenson R.H., Diercks D.B., Henderson S., Hanson K., Li S.Y., deFilippi C.R. (2011). Prognostic utility of ST2 in patients with acute dyspnea and preserved left ventricular ejection fraction. Clin. Chem..

[20] Santhanakrishnan R., Chong J.P., Ng T.P., Ling L.H., Sim D., Leong K.T., Yeo P.S., Ong H.Y., Jaufeerally F., Wong R. (2012). Growth differentiation factor 15, ST2, high-sensitivity troponin T, and N-terminal pro brain natriuretic peptide in heart failure with preserved vs. reduced ejection fraction. Eur. J. Heart Fail..

[21] Santos A.B., Kraigher-Krainer E., Gupta D.K., Claggett B., Zile M.R., Pieske B., Voors A.A., Lefkowitz M., Bransford T., Shi V. (2014). Impaired left atrial function in heart failure with preserved ejection fraction. Eur. J. Heart Fail..

[22] Dmour B.A., Miftode R.S., Iliescu Halitchi D., Anton-Paduraru D.T., Iliescu Halitchi C.O., Miftode I.L., Mitu O., Costache A.D., Stafie C.S., Costache I.I. (2021). Latest Insights into Mechanisms behind Atrial Cardiomyopathy: It Is Not always about Ventricular Function. Diagnostics.

[23] Xanthakis V., Larson M.G., Wollert K.C., Aragam J., Cheng S., Ho J., Coglianese E., Levy D., Colucci W.S., Michael Felker G. (2013). Association of novel biomarkers of cardiovascular stress with left ventricular hypertrophy and dysfunction: Implications for screening. J. Am. Heart Assoc..

[24] Weir R.A., Miller A.M., Murphy G.E., Clements S., Steedman T., Connell J.M., McInnes I.B., Dargie H.J., McMurray J.J. (2010). Serum soluble ST2: A potential novel mediator in left ventricular and infarct remodeling after acute myocardial infarction. J. Am. Coll. Cardiol..

[25] Shah R.V., Chen-Tournoux A.A., Picard M.H., van Kimmenade R.R., Januzzi J.L. (2009). Serum levels of the interleukin-1 receptor family member ST2, cardiac structure and function, and long-term mortality in patients with acute dyspnea. Circ. Heart Fail..

[26] Rehman S.U., Mueller T., Januzzi J.L. (2008). Characteristics of the novel interleukin family biomarker ST2 in patients with acute heart failure. J. Am. Coll. Cardiol..

[27] Dolapoglu A., Avci E., Yildirim T., Kadi H., Celik A. (2019). Using Soluble ST2 to Predict Adverse Postoperative Outcomes in Patients with Impaired Left Ventricular Function Undergoing Coronary Bypass Surgery. Medicina.

[28] Lotierzo M., Dupuy A.M., Cristol J.P., Roubille F., Cristol J.P. (2020). sST2 as a value-added biomarker in heart failure. Clin. Chim. Acta.

[29] Banaszkiewicz M., Pietrasik A., Darocha S., Piłka M., Florczyk M., Dobosiewicz A., Kędzierski P., Pędzich-Placha E., Kochman J., Opolski G. (2020). Soluble ST2 protein as a new biomarker in patients with precapillary pulmonary hypertension. Arch. Med. Sci..

[30] Bader F., Manla Y., Atallah B., Starling R.C. (2021). Heart failure and COVID-19. Heart Fail. Rev..

[31] Miftode E., Luca C., Manciuc C., Vâtă A., Hunea I., Miftode L., Bădescu A., Dorneanu O. (2020). Covid-19: A Course Through Stormy Waters. Med. -Surg. J..

[32] Grillet F., Behr J., Calame P., Aubry S., Delabrousse E. (2020). Acute Pulmonary Embolism Associated with COVID-19 Pneumonia Detected with Pulmonary CT Angiography. Radiology.

[33] Park J.F., Banerjee S., Umar S. (2020). In the eye of the storm: The right ventricle in COVID-19. Pulm. Circ..

[34] Pascual-Figal D.A., Pérez-Martínez M.T., Asensio-Lopez M.C., Sanchez-Más J., García-García M.E., Martinez C.M., Lencina M., Jara R., Januzzi L.J., Lax A. (2018). Pulmonary Production of Soluble ST2 in Heart Failure. Circ. Heart Fail..

[35] Murphy S.P., Kakkar R., McCarthy C.P., Januzzi J.L. (2020). Inflammation in Heart Failure: JACC State-of-the-Art Review. J. Am. Coll. Cardiol..

[36] Cao Z., Jia Y., Zhu B. (2019). BNP and NT-proBNP as Diagnostic Biomarkers for Cardiac Dysfunction in Both Clinical and Forensic Medicine. Int. J. Mol. Sci..

[37] Maisel A.S., Richards A.M., Pascual-Figual D., Mueller C. (2015). Serial ST2 testing in hospitalized patients with acute heart failure. Am. J. Cardiol..

[38] Huang F.Y., Wang H., Huang B.T., Liu W., Peng Y., Zhang C., Xia T.-L., Wang P.-J., Zuo Z.-L., Heng Y. (2016). The influence of body composition on the N-terminal pro-B-type natriuretic peptide level and its prognostic performance in patients with acute coronary syndrome: A cohort study. Cardiovasc. Diabetol.

[39] Tsutamoto T., Sakai H., Yamamoto T., Nakagawa Y. (2019). Renal Clearance of N-Terminal pro-Brain Natriuretic Peptide Is Markedly Decreased in Chronic Kidney Disease. Circ. Rep..

[40] Bayes-Genis A., Zamora E., de Antonio M., Galán A., Vila J., Urrutia A., Díez C., Coll R., Altimir S., Lupón J. (2013). Soluble ST2 serum concentration and renal function in heart failure. J. Card. Fail..

[41] Ruggiero A., Villa C.H., Bander E., Rey D.A., Bergkvist M., Batt C.A., Manova-Todorova K., Deen W.M., Scheinberg D.A., McDevitt M.R. (2010). Paradoxical glomerular filtration of carbon nanotubes. Proc. Natl. Acad. Sci. USA.

[42] Grande D., Leone M., Rizzo C., Terlizzese P., Parisi G., Gioia M.I., Leopizzi T., Segreto A., Guida P., Romito R. (2017). A Multiparametric Approach Based on NT-proBNP, ST2, and Galectin3 for Stratifying One Year Prognosis of Chronic Heart Failure Outpatients. J. Cardiovasc. Dev. Dis..

[43] Boulogne M., Sadoune M., Launay J., Baudet M., Cohen-Solal A., Logeart D. (2017). Inflammation versus mechanical stretch biomarkers over time in acutely decompensated heart failure with reduced ejection fraction. Int. J. Cardiol..

[44] Wettersten N., Maisel A. (2016). Biomarkers for Heart Failure: An Update for Practitioners of Internal Medicine. Am. J. Med..

[45] Biasucci L.M., Maino A., Grimaldi M.C., Cappannoli L., Aspromonte N. (2021). Novel Biomarkers in Heart Failure: New Insight in Pathophysiology and Clinical Perspective. J. Clin. Med..

[46] Gagging H.K., Motiwala S., Bhardwaj A., Parks K.A., Januzzi J.L. (2013). Soluble concentrations of the interleukin receptor family member ST2 and beta-blocker therapy in chronic heart failure. Circ. Heart Fail..

[47] Januzzi J.L., Pascual-Figal D., Daniels L.B. (2015). ST2 testing for chronic heart failure therapy monitoring: The International ST2 Consensus Panel. Am. J. Cardiol..

[48] Manzano-Fernández S., Januzzi J.L., Pastor-Pérez F.J., Bonaque-Gonzales J.C., Boronat-Garcia M., Pascual-Figal D.A. (2012). Serial monitoring of soluble interleukin family member ST2 in patients with acutely decompensated heart failure. Cardiology.

[49] Breidthardt T., Balmelli C., Twerenbold R., Mosimann T., Espinola J., Haaf P., Thalmann G., Moehring B., Mueller M., Meller B. (2013). Heart failure therapy-induced early ST2 changes may offer long-term therapy guidance. J. Card. Fail..

[50] Aleksova A., Paldino A., Beltrami A.P., Padoan L., Iacoviello M., Sinagra G., Emdin M., Maisel A.S. (2019). Cardiac Biomarkers in the Emergency Department: The Role of Soluble ST2 (sST2) in Acute Heart Failure and Acute Coronary Syndrome-There is Meat on the Bone. J. Clin. Med..

[51] Lassus J., Gayat E., Mueller C., Peacock W.F., Spinar J., Harjola V.P., van Kimmenade R., Pathak A., Mueller T., Disomma S. (2013). Incremental value of biomarkers to clinical variables for mortality prediction in acutely decompensated heart failure: The Multinational Observational Cohort on Acute Heart Failure (MOCA) study. Int. J. Cardiol..

